# Methodology for the Generation of High-Quality Ultrasonic Images of Complex Geometry Pieces Using Industrial Robots

**DOI:** 10.3390/s23052684

**Published:** 2023-03-01

**Authors:** Sofía Aparicio Secanellas, Iñaki Gauna León, Montserrat Parrilla, Montserrat Acebes, Alberto Ibáñez, Héctor de Matías Jiménez, Óscar Martínez-Graullera, Alberto Álvarez de Pablos, Margarita González Hernández, José Javier Anaya Velayos

**Affiliations:** 1 Institute for Physical and Information Technologies “Leonardo Torres Quevedo”, ITEFI, Spanish National Research Council (CSIC), 28006 Madrid, Spain; 2 Tecnitest Ingenieros SL, 28021 Madrid, Spain

**Keywords:** industrial robots, complex geometry pieces, ultrasonic images, synchronism

## Abstract

Industrial robotic arms integrated with server computers, sensors and actuators have revolutionized the way automated non-destructive testing is performed in the aeronautical sector. Currently, there are commercial, industrial robots that have the precision, speed and repetitiveness in their movements that make them suitable for use in numerous non-destructive testing inspections. Automatic ultrasonic inspection of complex geometry parts remains one of the most difficult challenges in the market. The closed configuration, i.e., restricted access to internal motion parameters, of these robotic arms makes it difficult for an adequate synchronism between the movement of the robot and the acquisition of the data. This is a serious problem in the inspection of aerospace components, where high-quality images are necessary to assess the condition of the inspected component. In this paper, we applied a methodology recently patented for the generation of high-quality ultrasonic images of complex geometry pieces using industrial robots. The methodology is based on the calculation of a synchronism map after a calibration experiment and to introduce this corrected map in an autonomous, independent external system developed by the authors to obtain precise ultrasonic images. Therefore, it has been shown that it is possible to establish the synchronization of any industrial robot with any ultrasonic imaging generation system to generate high-quality ultrasonic images.

## 1. Introduction

The aeronautical sector is a strategic sector that has been subjected to great activity since its inception and, more specifically, in recent years since it is in an international context marked by major changes in the relationships within the value chain and a complete globalization of the market with new opportunities for growth and activity.

The constant need to overcome the technological barriers that mark the aeronautical sector poses new challenges in the short-, medium- and long-term. In this context, the Technological Platform of Advanced Materials and the Aerospace Spanish Platform have identified (thanks to a representative group of the main aeronautical components and structures manufacturers operating in Spain) the need for research, development and innovation in the field of materials in the short–medium-term (2020–2030).

Non-destructive inspection techniques, beyond the use as support in the manufacture of components, are extremely important in the industry [1]. In addition to enabling the introduction of new materials and/or designs in a sector such as aerospace, which has a clear incentive for technological developments, they have a very beneficial impact on design capabilities in other sectors [2,3].

Ultrasonic examination is one of the most suitable and easily automated methods for inspections of composite materials [4,5]. In contrast to other techniques, they have the advantage that the nature of both the matrix and the type of fibers do not affect the result of the inspection. They also have great importance in the defectology of composite materials because it is possible to go over great depths, the technique is quite fast, it has a very practical and simple use, and at the same time, it allows the detection of different defects and the knowledge of the material in different ways of failure [6].

However, despite being a widely used technique in the field of composite materials [7], inspections on critical parts or complex geometry entail additional difficulties compared to standard inspection, needing special requirements, both from the point of view of the inspection and the evaluation. These needs are common to different industrial sectors where high reliability, high precision and adequate cost control are required [8].

Cartesian robots are widely used in the aeronautical sector because it is easy to know the position of the transducer in the piece at each moment. However, every time the pieces to be inspected are more complex, and it is not possible to use Cartesian robots; therefore, complex industrial robots must be used. A good option for the inspection of parts with complex geometry is the use of robotic arms. Industrial robotic arms can manipulate and move ultrasonic probes along programmed paths with enough speed, precision, and repeatability to automate inspections. The main drawback of this approach is that the control systems of current industrial robots do not provide information on the position of their end effector with the precision and updating rate that the strict inspection standards for aerospace components require. Therefore, the inspection of parts with complex geometry, in which the movement of the ultrasonic transducer along the inspection trajectories is not carried out at a constant and known speed, and where it is not possible to install encoders that report the transducer position cannot be performed with commercial, industrial robots.

In [9], a scan path was generated offline for automated eddy currents non-destructive inspection of a complex shape test piece by means of a six-axis KUKA robotic arm. 

A dual-robot NDT system is established in [10] for the inspection of semi-enclosed workpieces and presents a specific trajectory planning approach for the NDT scheme to inspect these workpieces. 

To reduce the inspection time for evaluating defects in geometrically complex structures, a more efficient scheme in which the test object is grasped by the robot (TOGR) is investigated in [11]. In this work, a six-degrees-of-freedom industrial robot is used to hold the non-destructive testing (NDT) probe normal to the test surface, and ultrasonic time-domain reflectometry (UTDR) is used to identify the flaw echo. The ultrasonic signals are acquired synchronously with positional data regarding the robot. The TOGR solution shows a significant improvement in the positional accuracy of the robotic trajectory.

A new scanner path planning method for part inspection using an industrial six-axis robot is presented in [12]. The novelty of the approach is to generate a scan path with the control of the overlap between two adjacent scanning paths based on the use of the least-squares conformal maps, which stretches a 3D mesh surface on a 2D plane. 

In [13], a dual-robot air-coupled ultrasonic non-destructive testing scheme is shown and introduces the structure of the system and a general calibration method for the workpiece frame of a dual-robot system in detail. A tangential constraint method, which makes the probes completely aligned during the inspection process, is proposed.

In this paper, a synchronism methodology recently patented [14] is proven to facilitate the generation of ultrasonic images of pieces of complex geometry through the use of industrial robots. This methodology is based on a previous calibration method performed in the piece to be inspected, adding discontinuities in known positions to obtain the instants of time in which the acquisition system must emit an ultrasonic pulse. These instants of time are used by an external independent system to synchronize the movement of the robot with the ultrasonic system. Therefore, in this paper, we present an autonomous, independent external system whose information comes mainly from the generated ultrasonic images without acting on the programming of the robot. Moreover, the synchronization of any industrial robot with any system of generation of ultrasonic images will be established.

## 2. Materials and Methods

The methodology for the generation of high-quality ultrasonic images of complex geometry pieces using industrial robots is based on a calibration method to compute a more precise synchronism map. A synchronism map is a trigger synchronism that determines the instants of time in which the acquisition system must emit an ultrasonic pulsed wave to obtain pixel values to fulfill inspection requirements. The calibration method starts generating an initial synchronism map by carrying out a constant pulse repetition frequency (PRF) inspection of a standard specimen with discontinuities in known positions. After that, the initial synchronism map is processed to obtain a more precise map, and this new map is introduced in an autonomous, independent external system to synchronize the movement of the robot with the ultrasonic system.

We assume that the geometric variations when installing the different pieces of the same type in the inspection system and the variations of the trajectories described by the robot in repetitions of its program are, in all cases, lower than the desired resolution for the inspection. Therefore, if a map is built with the time elapsed from the start of each inspection line and the position of the transducers on the piece, this can be applied to the inspection of all pieces of the same type.

### 2.1. Inspection Systems

**Figure 1:**
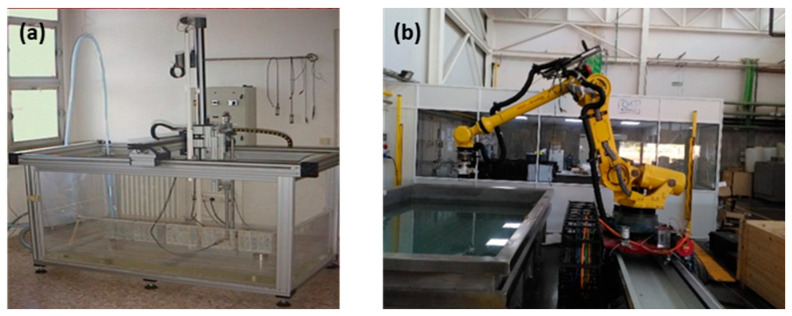
(**a**) Cartesian robot at ITEFI laboratory used for the initial tests. (**b**) Industrial robot used for the experiments at Tecnitest Company.

The methodology was proved first using a Cartesian robot available at ITEFI laboratory in Madrid, Spain, and after that, a Tecnitest Company in Madrid, Spain, industrial robot was used, see Figure 1.

A standard automatic system (with three Cartesian axes) was used to test the methodology at the Institute for Physical and Information Technologies “Leonardo Torres Quevedo” (ITEFI) laboratory.

The Tecnitest Company industrial robot used was the Fanuc Brand model R-2000 iC/125 L with 6 axes [15]. This robot is equipped with Socomate model SOCO-8S-UT- Multiplexer ultrasonic equipment [16].

### 2.2. Standard Specimen

A standard piece has been used to fine-tune the methodology that allows the position of the transducers to be accurately determined during the scan. Any standard specimen must have the following characteristics:

(1) They must generate discontinuities in the ultrasonic image that are easy to detect, ideally through automatic defect detection processes or at least through visual inspection by trained operators.
(2) The positions of the marks that produce the discontinuities must be known. The separation of these marks depends on the inspection areas where accelerations of robot occur and must allow detection of the maximum accelerations.

**Figure 2:**
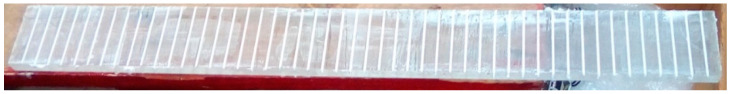
Plastic specimen fabricated at ITEFI laboratory.

A standard plastic specimen with 47 slots 3 mm wide and separated 10 mm was fabricated at ITEFI laboratory, Figure 2.

**Figure 3:**
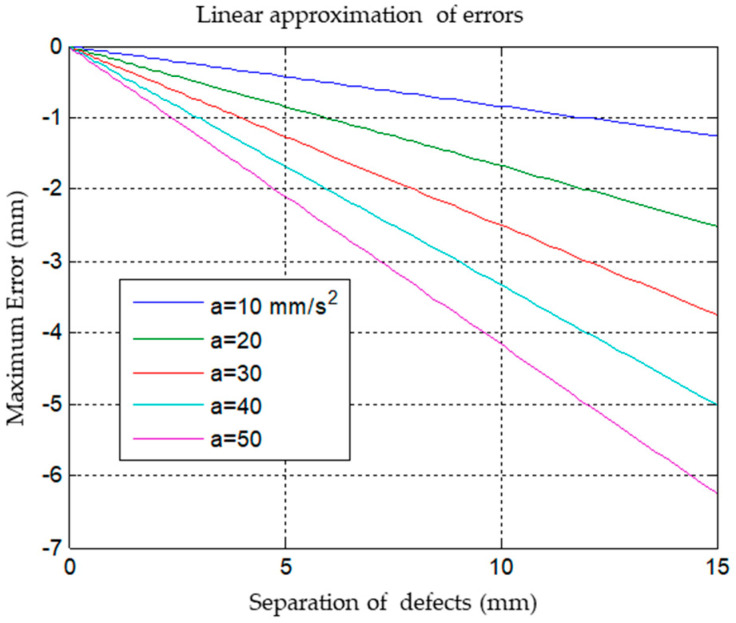
Linear approximation of errors depending on the separation of defects of the test specimen.

Suppose the accelerations are uniform, in general. In that case, three defects are required in the acceleration zones so that the error in determining the position of the transducer is almost zero when performing an adjustment using a cubic function. With this specimen, it was verified that if three defects are not available and a linear adjustment is made, the position error will depend on the acceleration value, as can be seen in the following graph, see Figure 3. This figure shows the error depending on the separation of defects in the piece to be inspected and the acceleration of the robot’s movement. If we know the maximum acceleration of the robot (we usually know), we can determine the separation of the defects to ensure a maximum error of positioning.

### 2.3. Sync Map Generation

The synchronism map is obtained from an inspection of the standard piece carried out at constant PRF that allows us to determine the position of the transducers from the marks of the standard specimen. Depending on the type of inspection, the process can be more or less complex. In a rectangular scan, it will be enough to correct the comb that usually occurs in any automatic inspection system. While in complex scans, where neither the trajectories nor the speeds are the same, each scan line will have to be calibrated, and the trigger instants corresponding to each image line must be generated.

#### 2.3.1. Images Filtering

**Figure 4:**
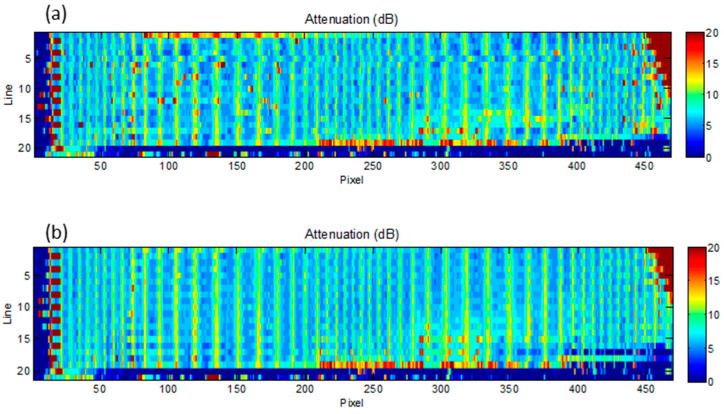
Ultrasonic image of the plastic specimen using water jets as coupling method. (**a**) original image. (**b**) image after filtering.

To facilitate the detection of discontinuities in the pattern piece, we used a usual process to remove pixels out of range. These pixels are created in the image for various reasons; the most common are the drops that are produced when inspections are carried out by coupling with water jets, see Figure 4a. Impulsive electromagnetic noises are also usually frequent in industrial environments. These pixels must be removed since; otherwise, they generate large errors in the automatic detection of defects. The usual techniques to eliminate these noises are based on the redundancy of the images, either by averaging or by using nonlinear filters such as the median filter, Figure 4b.

#### 2.3.2. Determination of the Acquisition Instants

**Figure 5:**
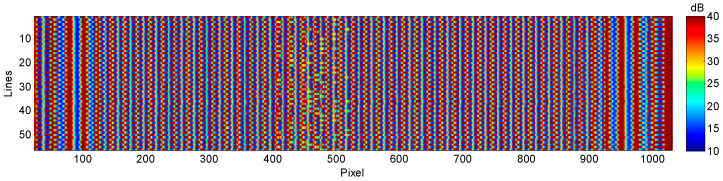
Ultrasonic image of the plastic specimen obtained with the Cartesian robot at ITEFI laboratory.

Figure 5 represents the ultrasonic image of the specimen obtained at constant PRF. In this case, a rectangular scan has been carried out with the ITEFI Cartesian robot, and it can be observed that the main distortion in the image is due to the comb. In this case, it would suffice to generate a time map for the even lines and another for the odd lines that would be repeated throughout the entire scan.

**Figure 6:**
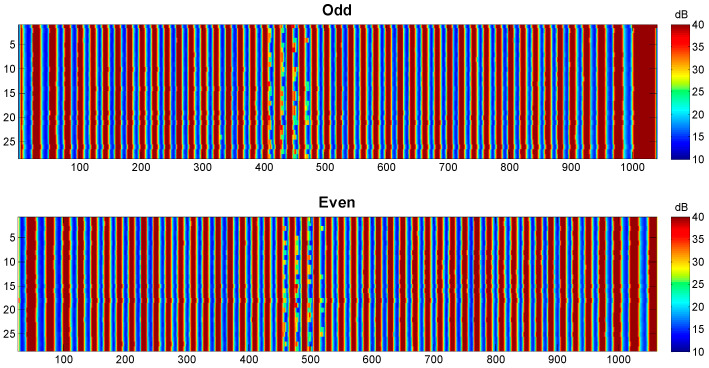
Ultrasonic image of the plastic specimen divided by odd and even lines obtained with the Cartesian robot at ITEFI laboratory.

Therefore, the image obtained is divided into two, one formed by odd lines and the other by even lines, see Figure 6.

The correction of the comb would be carried out by measuring the displacement between two equivalent areas of the specimen and adding an initial delay equivalent to half the number of pixels of the displacement. For example, a pulse repetition period of 25 ms (PRF = 40 Hz) was used to obtain this image; the displacement of the specimen center lines is 48 pixels; therefore, the initial delay in applying to the odd and even lines is 24 × 25 ms = 600 ms.

**Figure 7:**
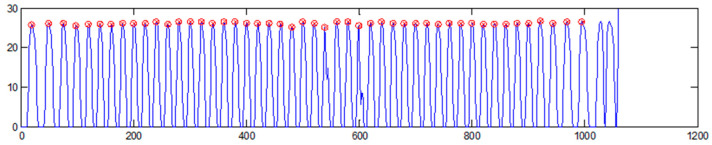
Maximum detection of the discontinuities of the plastic specimen after the convolution.

To determine the position of the discontinuities of the specimen in the image obtained, we convolved each line of the image with the shape of the discontinuities used (in this case, a rectangular pulse). The result of this processing is an image with the maxima located at the position of the defects. This maximum can be detected with any maximum relative detector, but a manual correction of the position of some of the defects will often be necessary if the image of the defects is not of sufficient quality. In the example in Figure 7, you can check the maximum displacement in the detection of some of the central grooves that were not sufficiently sharp.

From these measurements, N values $\left(X_{n},\;T_{n}\right)$, corresponding to the N reference marks, are obtained for each line, being $X_{n}$ the position of the mark and $T_{n}$ the time in which it appears is calculated with the following formula:

$$T_{n}=\frac{1}{PRF}\cdot k_{n}\;con\;n=\left\{1,\ldots ,N\right\}$$

being $k_{n}$ the position in pixels of the *n*-th mark and PRF the repetition frequency of pulses.

**Figure 8:**
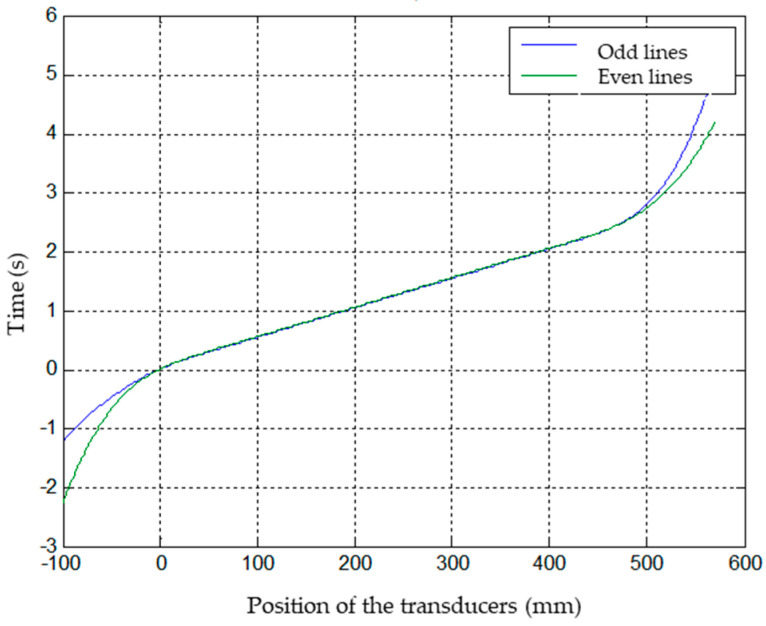
Position of the tranducers at each time for odd and even lines.

The result of this processing on the standard specimen is shown in Figure 8. As it is a rectangular scan, it is enough to distinguish between even and odd lines, having the position and time of the first defect of the specimen as the origin of coordinates.

**Figure 9:**
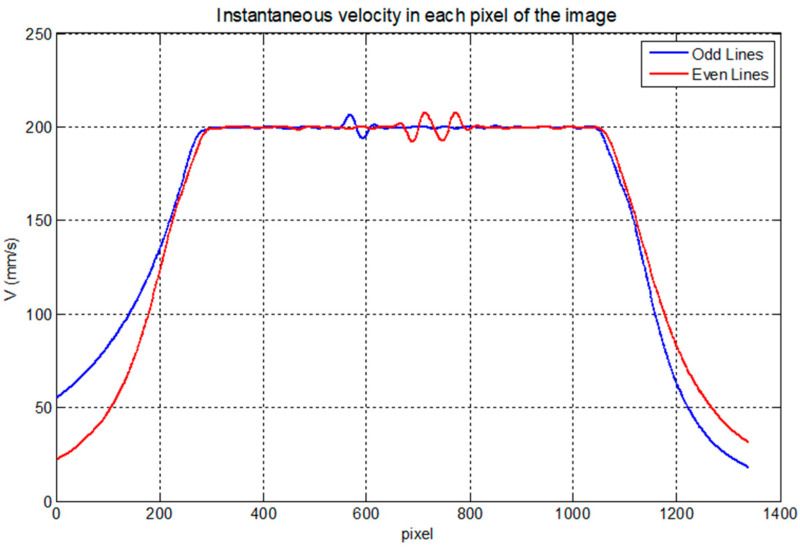
Velocity measured in each pixel of the image with the Cartesian robot at ITEFI laboratory.

From this processing, we can calculate different movement parameters of the inspection, such as the velocity, as shown in Figure 9. In this figure, you can see a typical movement of a linear inspection of flat pieces, an acceleration ramp until the programmed inspection speed is obtained, 200 mm/s, and then another deceleration ramp that is the same for all the lines.

Small oscillations can be observed when the robot goes at maximum speed. If these oscillations were greater, they could deteriorate the image obtained. It was verified that they are less than 5% of the maximum velocity, see Figure 9; therefore, they would give an error of 5% of the position of that pixel with respect to the previous one. Therefore, this error is completely negligible since the digitization of the ultrasonic image allows a precision of one pixel. 

With this methodology, we obtain a synchronized map to introduce in the external synchronization system to perform the corrected image of the piece.

### 2.4. External Synchronization System (ESS)

An autonomous, independent external system called iTotalSynchro was designed and developed by the authors. It is a programmable trigger pulse system that allows synchronizing the movement of the robot with the ultrasound system using the synchronism map generated in the calibration.

**Figure 10:**
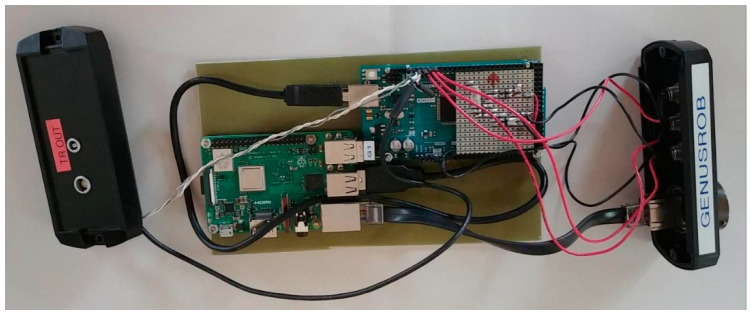
iTotalSynchro hardware composition.

The hardware of iTotalSynchro is composed of a Raspberry Pi 3b+ and an Arduino Mega 2560 mote with an SPI memory 23LC1024. The Arduino is connected by USB to the Raspberry, Figure 10. The Raspberry has been adapted with an external real-time clock (RTC) since it does not have an internal one.

The Raspberry oversees the communications between the robot and the Arduino. The Arduino mote will receive the information from the Raspberry, and it will activate a pin to tell the ultrasonic system to start the emission of pulses. For that purpose, server/client communication has been created to communicate the robot (client) with the server (Raspberry) using an Ethernet cable. Six different ultrasonic emission modes were added:

1. Positive edge.
2. Negative edge.
3. Right encoder.
4. Left encoder.
5. This mode uses an external hardware signal. Right encoder as mode 3 when an external trigger arrives.
6. This mode uses an external hardware signal. Left encoder as mode 4 when an external trigger arrives.

**Figure 11:**
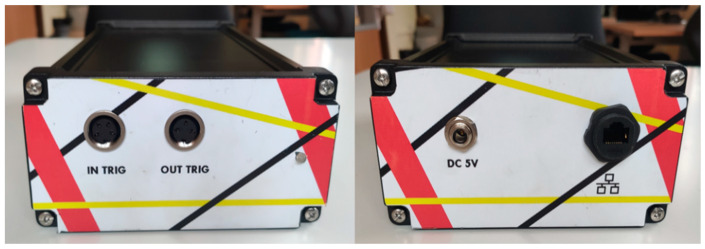
iTotalSynchro frontal vision.

The ability to shoot at constant PRF has also been considered. The Raspberry with the Arduino mote was introduced in an IP67 box to be suitable to place in the industrial robot. In Figure 11, the frontal vision of both sides of the synchronization system is shown. On the left side, input and output trigger connectors and a tricolor led can be observed to indicate to the user the different states of the system. On the right side, the power supply and the Ethernet connectors can be seen.

## 3. Results and Discussion

**Figure 12:**
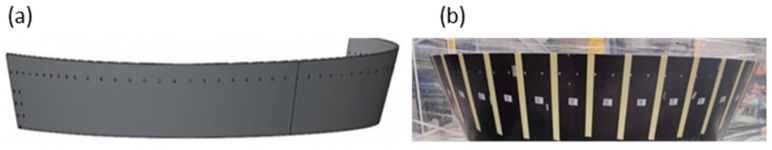
Complex piece to be inspected (**a**) plan of the piece and (**b**) real piece with the equidistant marks added to calibrate the movement.

**Figure 13:**
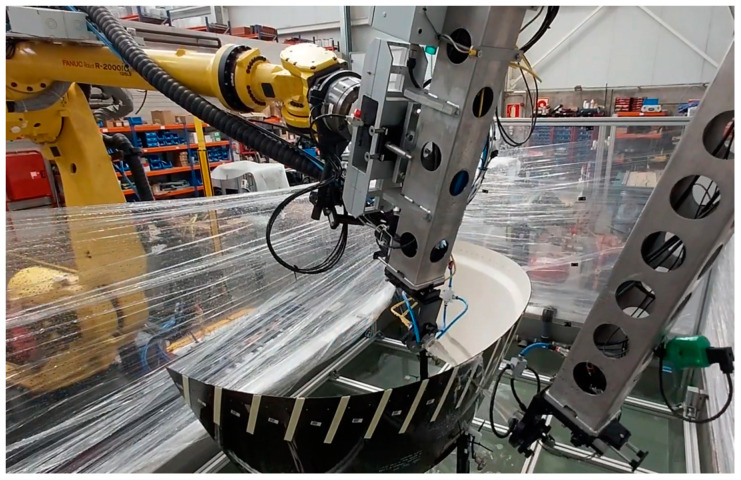
Assembly of the complex piece to be inspected for the experiments on the Industrial robot at Tecnitest Company.

In this section, the results obtained from applying the proposed methodology and using the external system developed by the authors in a real case are shown. For that purpose, a complex piece from Tecnitest Company was selected and inspected using the industrial robot that they have in their laboratory, Figure 12 and Figure 13.

In this case, to obtain the position of the transducers with respect to the piece during the scan, the same target piece was used as a pattern. A series of elements were incorporated that generate detectable discontinuities or uniform bands in the ultrasonic images and whose separation is known and constant, Figure 12b.

**Figure 14:**
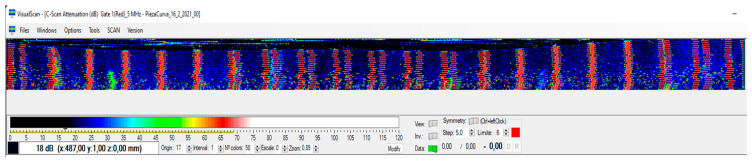
Imprecise image obtained at constant PRF.

As can be seen in Figure 14, the image obtained at constant PRF is distorted. This is due to the fact that the position of the acquisition module is not exactly known at each instant. The equidistant parallel bands incorporated into the piece are not obtained, they present irregularities, and most importantly, it would be difficult to evaluate the internal structure of the piece, which is the objective of this type of ultrasonic image.

**Figure 15:**
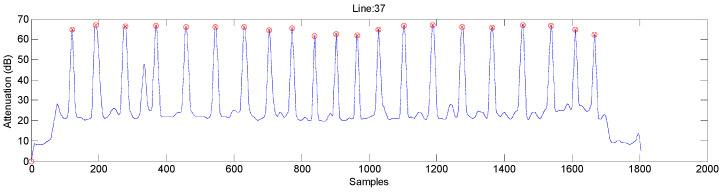
Band detection example.

From this image, the positions of the bands can be obtained, see Figure 15, and, based on them, the synchronism map can be generated to guarantee that the inspection is carried out in compliance with the demanded requirements and that it can be correctly evaluated. The method has been described in the previous section and is registered in the patent [14].

**Figure 16:**
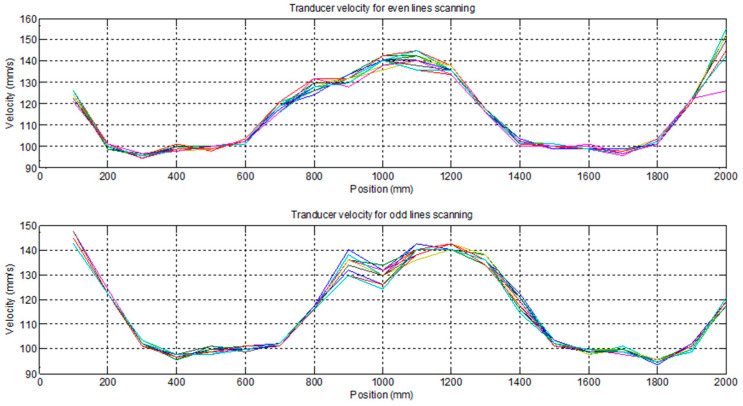
Velocity measured in each pixel of the image.

If we observe, in this case, the instantaneous velocity curves of the robot movement during the scan, Figure 16, we will see that they are not simple acceleration and deceleration ramps. They vary along the line, and they are different for each line scanned due to the conical shape of the piece. Differences are also observed in the movement of the robot between one and the other direction of movement.

**Figure 17:**
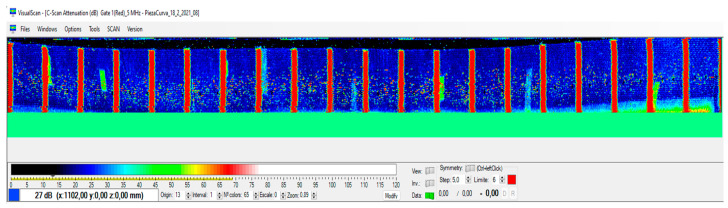
Final image obtained from the proposed methodology and using the external iTotalSynchro with the industrial robot.

Finally, applying the synchronism map through the iTotalSynchro external synchronization system, the image of Figure 17 is obtained. In this figure, the parallel bands incorporated into the piece can be observed, and therefore, it can be guaranteed that the inspection is carried out in compliance with the requirements demanded, and it will be possible to evaluate correctly.

Therefore, it becomes clear that it is possible to calibrate in an economical, precise and efficient way the trajectories followed by the displacement module in order to adequately synchronize the firing of each ultrasonic wave with the movement of the displacement module.

In this development, it has been shown that it is possible to establish the synchronization of any industrial robot with any ultrasonic imaging generation system by means of the developed synchronization module. Therefore, this system allows not depending on a specific robot and ultrasonic acquisition system, but rather it will be possible to choose the set that best suits the needs at any given time.

## 4. Conclusions

In this paper, a methodology recently patented [14] to obtain precise ultrasonic information on complex pieces using industrial robots is shown. For Cartesian robots, it is easy to know the position of the transducer in the piece at each moment. Every time the pieces to be inspected are more complex, it is not possible to use Cartesian robots; therefore, complex industrial robots must be used. For these robots, it is very difficult to know the position of the transducer in the inspected piece, and they are closed systems, i.e., there is no access to their configuration. To solve this problem, we propose a methodology to overcome these difficulties that have been patented. We apply this methodology to a complex aeronautical piece and show the results obtained. The methodology is based on the calculation of a corrected synchronism map after a calibration experiment to introduce this corrected map in an external system developed by the authors to obtain precise ultrasonic images. Therefore, it has been shown that it is possible to establish the synchronization of any industrial robot with any ultrasonic imaging generation system by means of the developed synchronization module.

In addition, the validity of the described procedure has been demonstrated both in simple geometry parts, such as the rectangular specimen tested at the ITEFI facilities with a Cartesian robot, and in more complex pieces, such as the one inspected at Tecnitest using a robotic arm.

## 5. Patents

The methodology explained and proved in this article has been patented and licensed by Tecnitest Company [9].

## Data Availability

Not applicable.
